# Proteomic, mechanical, and biochemical development of tissue-engineered neocartilage

**DOI:** 10.1186/s40824-022-00284-4

**Published:** 2022-07-22

**Authors:** Benjamin J. Bielajew, Ryan P. Donahue, Elliott K. Lamkin, Jerry C. Hu, Vincent C. Hascall, Kyriacos A. Athanasiou

**Affiliations:** 1 Department of Biomedical Engineering, University of California Irvine, Irvine, CA USA; 2 Department of Biomedical Engineering, Cleveland Clinic, Cleveland, OH USA

**Keywords:** Cartilage, Cartilage tissue engineering, Proteomics, Development, Knee cartilage

## Abstract

**Background:**

The self-assembling process of cartilage tissue engineering is a promising technique to heal cartilage defects, preventing osteoarthritic changes. Given that chondrocytes dedifferentiate when expanded, it is not known if cellular expansion affects the development of self-assembled neocartilage. The objective of this study was to use proteomic, mechanical, and biochemical analyses to quantitatively investigate the development of self-assembled neocartilage derived from passaged, rejuvenated costal chondrocytes.

**Methods:**

Yucatan minipig costal chondrocytes were used to create self-assembled neocartilage constructs. After 1, 4, 7, 14, 28, 56, or 84 days of self-assembly, constructs were analyzed through a variety of histological, biomechanical, biochemical, and proteomic techniques.

**Results:**

It was found that temporal trends in neocartilage formation are similar to those seen in native hyaline articular cartilage development. For example, between days 7 and 84 of culture, tensile Young’s modulus increased 4.4-times, total collagen increased 2.7-times, DNA content decreased 69.3%, collagen type II increased 1.5-times, and aggrecan dropped 55.3%, mirroring trends shown in native knee cartilage. Importantly, collagen type X, which is associated with cartilage calcification, remained at low levels (≤ 0.05%) at all neocartilage developmental time points, similar to knee cartilage (< 0.01%) and unlike donor rib cartilage (0.98%).

**Conclusions:**

In this work, bottom-up proteomics, a powerful tool to interrogate tissue composition, was used for the first time to quantify and compare the proteome of a developing engineered tissue to a recipient tissue. Furthermore, it was shown that self-assembled, costal chondrocyte-derived neocartilage is suitable for a non-homologous approach in the knee.

**Supplementary Information:**

The online version contains supplementary material available at 10.1186/s40824-022-00284-4.

## Introduction

Focal cartilage defects occur in 12% of the population [1] and 36% of athletes [2], and cartilage defects are well-known not to heal. Existing surgical procedures to address focal cartilage defects, such as microfracture or matrix-assisted autologous chondrocyte implantation, only provide short-term relief [3, 4]. Focal cartilage defects can eventually degenerate to osteoarthritis (OA) [5], which affects over 32.5 million adults in the US [6]. Tissue engineering holds promise for regenerating cartilage defects by alleviating pain, restoring function, and preventing the onset of OA [3, 7, 8]. For successful translation of tissue-engineered cartilages from the laboratory to human usage, neocartilages must be well-characterized for quality control with appropriate release criteria for preclinical and clinical trials. The quality and safety profile of any implant will benefit greatly from the ability to quantitatively define the implant’s composition.

Toward the precise determination of tissue composition for quality control and release criteria of tissue-engineered implants, it is desirable to quantify many analytes in a single sample with low sample volume. The advent of powerful quantitative bottom-up proteomics techniques [9] enables the simultaneous quantification of hundreds of proteins in biological samples, for example, in cartilage extracellular matrix (ECM). This engenders tissue engineers to establish new quality control protocols, where bottom-up proteomics can be used as a basis for a multitude of release criteria. Applications of bottom-up proteomics to investigate tissue composition is applicable to any neotissue that has ECM-dependent functionality (e.g., cartilage, skin, tendon/ligament, heart valve). Moreover, quantitative bottom-up proteomics can be used as a tool to interrogate developmental changes in neotissues, such as in tissue-engineering approaches for hyaline articular cartilage.

Self-assembled neocartilage derived from primary articular chondrocytes matures similarly to the way that native cartilage develops (i.e., it mimics aspects of mesenchymal condensation) [10]. Additionally, self-assembled neocartilages have been produced with native-like mechanical properties, such as a tensile modulus of 8.4 MPa [11] and an aggregate modulus of 400 kPa [12]; these robust mechanical properties are crucial for implant survival and functionality [13]. However, harvesting primary articular chondrocytes, which were used in these neocartilages, can lead to donor site morbidity or yield cells with an osteoarthritic phenotype [14]. Costal cartilage has shown potential as an alternative cell source in autologous and allogeneic approaches. Recent in vitro studies have shown the similar mechanical and biochemical attributes of rib cartilage and articular cartilage, deeming costal cartilage a valuable cell source for articular cartilage tissue engineering [15]. Additionally, self-assembled neocartilages have recently been made from extensively passaged, rejuvenated costal chondrocytes [16, 17], allowing for thousands of robust neocartilage implants to be made from one rib cartilage biopsy [18]. Self-assembled neocartilage implants made with costal chondrocytes have also been used to treat cartilage defects in Yucatan minipig models [19]. Transplantation and autologous chondrocyte implantation procedures in the clinic have also been performed [20]. For example, recent first-in-human efforts include using costal chondrocytes in a pellet-type autologous chondrocyte implantation to treat full-thickness lesions in knee articular cartilage [21]. Despite these promising advances in preclinical and clinical tissue engineering strategies using costal chondrocytes, it is not known whether self-assembled neocartilage made with costal chondrocytes is suitable for non-homologous implantation into the knee. Given that expanded chondrocytes undergo dedifferentiation – they quickly lose their chondrogenic phenotype [22–26] – it is important to determine whether self-assembled neocartilage produced from expanded chondrocytes develops similarly to native cartilage.

The objective of this study is to determine, through mechanical, biochemical, and proteomic analyses, whether neotissues formed from passaged, rejuvenated, and self-assembled costal chondrocytes display features of the native hyaline cartilage developmental process. The study design compares multiple protein analytes throughout maturation of self-assembled neocartilage, thereby informing how ECM components form mechanically robust tissue. The hypothesis of this work is that self-assembled neocartilage derived from passaged, rejuvenated costal chondrocytes will follow temporal trends in mechanical, biochemical, and proteomic properties that have previously been characterized in native hyaline cartilage development [27]. Specifically, as the self-assembled neocartilage develops from nascent tissue to mature neocartilage, it is expected that, 1) in mechanics, tensile and compressive properties will increase, 2) in biochemical composition, collagen content will increase, and glycosaminoglycan (GAG) and DNA content will decrease, and 3) in proteomics, collagen type II will increase, aggrecan and link protein will decrease, and, unlike in native costal cartilage, collagen type X will only be deposited at low levels (< 0.1%). This work will further the understanding of how the self-assembling process mimics native cartilage development and will determine the suitability of costal chondrocyte-derived neocartilage for non-homologous implantation into the knee.

## Methods

### Costal cartilage harvest and isolation

**Fig. 1:**
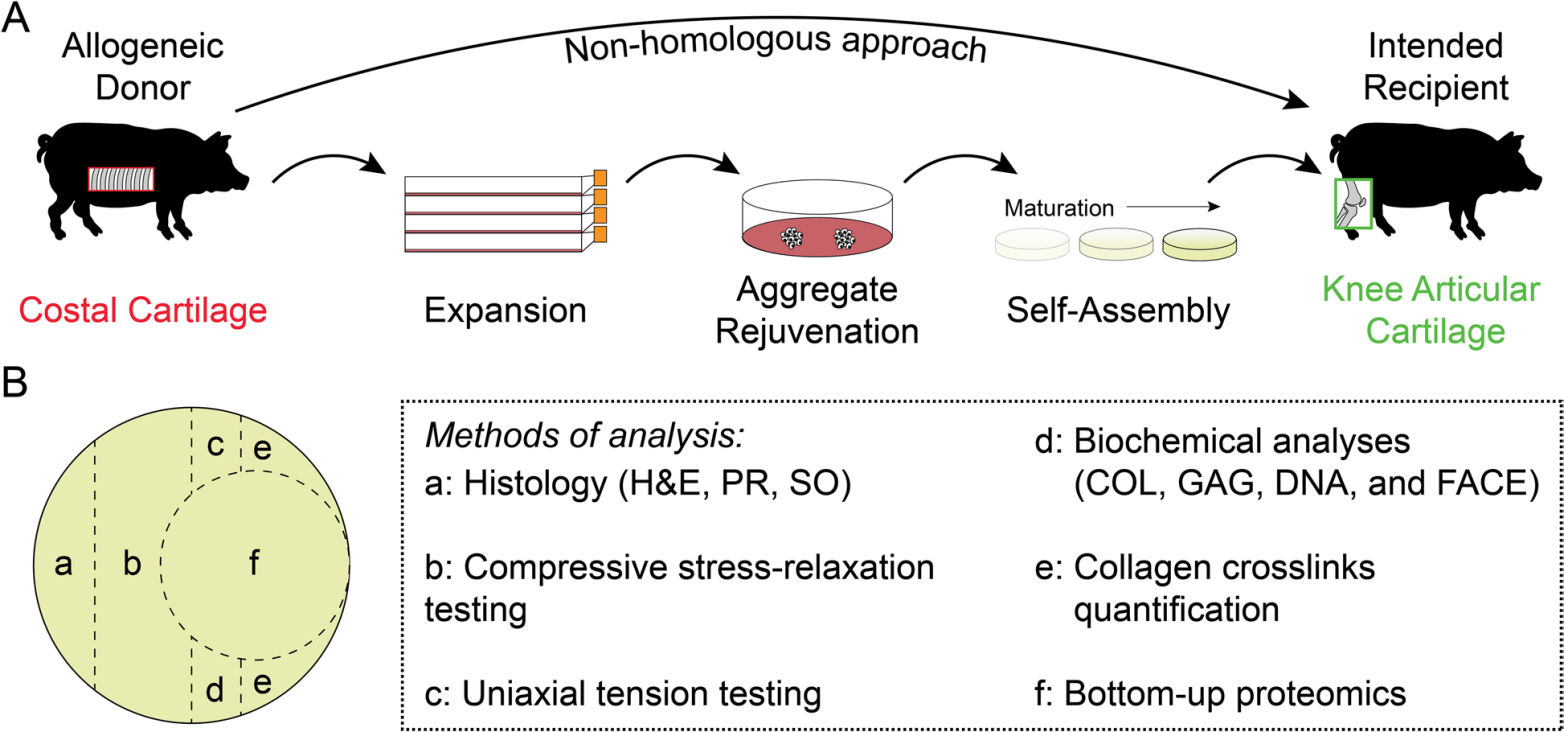
The tissue-engineering process using costal chondrocytes and neocartilage sample preparation. (**A**) Self-assembled constructs are envisioned to be used in the knee through an allogeneic, non-homologous approach. Donor costal chondrocytes were expanded, rejuvenated, and self-assembled in vitro for eventual implantation into the knee. (**B**) At specific maturation time points (i.e., days of culture), the neocartilage constructs were divided and assayed by various methods. Histological analysis included staining with hematoxylin and eosin (H&E), picrosirius red (PR), and safranin O with fast green counterstains (SO). Mechanical analysis included uniaxial tensile testing and compressive stress-relaxation testing. Biochemical analyses for total collagen (COL), glycosaminoglycan (GAG), and DNA content and fluorophore assisted carbohydrate electrophoresis (FACE) were performed from a papain digest

Costal chondrocytes were harvested from the rib cartilage of three juvenile (aged 5–8 months) Yucatan minipig donors that were culled for reasons unrelated to this study (Fig. 1A). Briefly, using sterile tools in a biosafety cabinet, costal cartilage was exposed, and the perichondrium was removed. Then, costal cartilage was minced to approximately 1 mm^3^ pieces and digested at 37 °C in 0.4% w/v pronase for 1 h followed by 0.2% w/v collagenase for 18 h. Both enzymes were dissolved in Dulbecco’s modified Eagle’s medium (DMEM, high glucose, GlutaMAX supplement) supplemented with 3% fetal bovine serum (FBS) and 1% penicillin–streptomycin-fungizone (PSF). The resulting cell suspension was filtered through a 70 µm cell strainer and treated with ammonium-chloride-potassium lysis buffer, as previously described [28].

### Costal chondrocyte expansion and aggregate rejuvenation

After isolation, costal chondrocytes were plated at 2.5 M cells per T225 flask (~ 11,111 cells/cm^2^) in chemically defined chondrogenic (CHG) medium (Fig. 1A), which consisted of DMEM, 1% PSF, 1% nonessential amino acids, 1% insulin-transferrin-selenous acid + , 100 nM dexamethasone, 50 µg/mL ascorbate-2-phosphate, 40 µg/mL L-proline, and 100 µg/mL sodium pyruvate. CHG medium was further supplemented with 2% FBS, 1 ng/mL transforming growth factor beta 1 (TGF-β1), 5 ng/mL basic fibroblast growth factor (bFGF), and 10 ng/mL platelet-derived growth factor (PDGF) during monolayer expansion to retain post-expansion chondrogenic potential [29]. Medium was changed every 3–4 days during expansion. After one passage, chondrocytes were frozen in FBS containing 10% dimethyl sulfoxide (DMSO) for downstream use. Cells were thawed and plated in CHG medium containing FBS and growth factors, as described above. Between passages, cells were lifted using 0.05% trypsin–EDTA solution for 9 min, and the cell sheet was digested using 0.2% w/v collagenase in DMEM containing 3% FBS and 1% PSF for approximately 30 min with agitation every 10 min. After six passages, the expanded cells underwent aggregate rejuvenation for 14 days to return them to a chondrogenic phenotype (Fig. 1A), as previously described [30]. Medium was changed every 3–4 days during aggregate rejuvenation. Cells were cultured in CHG medium supplemented with 10 ng/mL TGF-β1, 100 ng/mL growth differentiation factor 5 (GDF-5), and 100 ng/mL bone morphogenetic protein 2 (BMP-2). Aggregates were then digested after culture in 0.05% trypsin–EDTA solution for 45 min followed by 0.2% w/v collagenase solution in DMEM supplemented with 3% FBS and 1% PSF for 90 min with agitation every 10 min. The resulting cell suspension was filtered through a 70 µm cell strainer prior to the self-assembling process.

### Neocartilage self-assembly and bioactive factor treatment

As previously described [18], scaffold-free neocartilage self-assembly was carried out for a total of 84 days (Fig. 1A). Briefly, nonadherent wells of 5 mm diameter in size were made using molten 2% agarose and negative molds. The wells were hydrated with CHG medium, and the medium was changed at least three times prior to cell seeding. As previously optimized [31], 2 M cells per well were seeded in 100 µL CHG medium. After 4 h, medium was topped off with an additional 400 µL CHG medium, and, subsequently, medium was exchanged every day until day 3 when constructs were unconfined from agarose wells. From day 3 onward, neocartilage constructs were fed 2 mL CHG medium every 2 days. Constructs were treated with bioactive factors, as previously described [18]. Briefly, TGF-β1 (10 ng/mL) was supplemented continuously in CHG medium. Chondroitinase ABC (c-ABC) was applied to constructs at 2 U/mL in 0.4 mL of CHG for 4 h on day 7 of self-assembly. C-ABC was activated with 50 mM of sodium acetate and quenched with 1 mM zinc sulfate. Lysyl oxidase-like 2 at was added to the medium 0.15 µg/mL with 0.146 mg/mL hydroxylysine and 1.6 µg/mL copper sulfate from day 7 until the end of self-assembly.

### Sample preparation

Self-assembled neocartilage constructs (*n* = 7–9 per time point) were removed from culture after 1, 4, 7, 14, 28, 56, or 84 days of culture and photographed. Constructs at days 1 and 4 of culture disintegrated upon handling, and, thus, were not able to be photographed. Constructs were cut with a biopsy punch and scalpel for histological, mechanical, biochemical, and proteomic analyses as depicted in Fig. 1B. Samples for photometric biochemical assays and crosslinks mass spectrometry were weighed to obtain the wet weights (WWs). WWs were not able to be taken on day 1 constructs because they disintegrated upon contact with the weigh boat. After at least 72 h of lyophilization, samples were reweighed to obtain dry weights (DWs). Hydration was calculated based on the ratio of sample DW to WW.

### Histology

As previously described [32], samples were fixed in 10% neutral buffered formalin for at least 72 h, processed, embedded in paraffin, sectioned to 5 µm thickness, and mounted on microscopy slides. Samples were then stained with hematoxylin and eosin (H&E), safranin O with fast green counterstain (SO), or picrosirius red (PR). Representative images were taken at 20 × magnification using a brightfield microscope.

### Mechanical testing

Mechanical properties of constructs were quantified with compressive stress-relaxation and uniaxial tension tests. Punches of 3 mm diameter from neocartilage constructs were subjected to compressive stress-relaxation testing. Because day 1 and day 4 constructs disintegrated upon handling, they were not included in the mechanical testing analysis. As previously described [33], the sample height was determined using a tare load of 0.1 N. Samples were subjected to 15 preloading cycles of 5% strain based on the determined sample height. Strains of 10% and 20% were applied to the punch and held for 600 and 900 s, respectively. The force–displacement curves were fit to a standard linear solid model using a custom MATLAB code to determine relaxation modulus, instantaneous modulus, and coefficient of viscosity for each strain level. For tensile testing, samples were trimmed into dog bone shapes (approximately 0.75 mm by 0.45 mm) and glued to paper tabs of a predefined gauge length (1.55 mm), as previously described [32]. Samples were pulled until failure at 1% strain per second. Force–displacement curves were analyzed using a custom MATLAB code to extract Young’s modulus and ultimate tensile strength (UTS).

### Collagen, GAG, and DNA assays

Construct pieces were subjected to overnight digestion with papain, followed by biochemical assays for quantification of total collagen (COL), GAG, and DNA contents, as previously described [34]. Briefly, COL was quantified using a modified hydroxyproline assay [35]. GAG was quantified using a dimethylmethylene blue assay kit, and DNA was quantified using a PicoGreen assay kit. The COL, GAG, and DNA measurements were normalized to WW and DW. Three technical replicates per sample were averaged and used in the hydroxyproline assay, dimethylmethylene blue assay, and PicoGreen assay.

### Fluorophore assisted carbohydrate electrophoresis (FACE)

Papain digest aliquots (50 μL) from each sample were lyophilized, and GAGs were precipitated with alcohol and digested with c-ABC. Chondroitin-6-sulfate (CS6) and chondroitin-4-sulfate (CS4) were derivatized using 2-aminoacridone, and CS6 was separated from CS4 using FACE, as previously described [36]. CS6 and CS4 were quantified by integrating the optical density of CS6 and CS4 bands in ImageJ, then comparing the resulting integrated optical density in samples and standards. CS6 was divided by CS4 to obtain the chondroitin sulfate ratio.

### Collagen crosslink quantification

Quantification of collagen crosslinks was performed, as previously described [37]. Briefly, construct pieces approximately 1 mg in WW were lyophilized, weighed, reduced in NaBH_4_, washed on a rocker plate overnight in ultrapure water, and hydrolyzed overnight in HCl. HCl was evaporated, hydrolysates were resuspended and filtered, and then hydrolysates were subjected to liquid chromatography-mass spectrometry with a Waters ACQUITY QDa quadrupole mass spectrometer to quantify mature pyridinoline (PYR), immature dihydroxylysinonorleucine (DHLNL), hydroxyproline (OHP), and internal standard pyridoxine. Because day 1 constructs disintegrated during the washing process, they were not included in the crosslinks analysis.

### Bottom-up proteomics

Bottom-up proteomics was performed, as previously described [37]. Three samples per group, chosen at random, were used for bottom-up proteomics. Briefly, construct pieces approximately 1 mg in WW were lyophilized, weighed, washed by vortexing twice in 10 mM ammonium citrate and twice in 50 mM ammonium bicarbonate, digested overnight in trypsin, and subjected to liquid chromatography-tandem mass spectrometry on a Thermo Fisher Scientific Orbitrap Fusion Lumos mass spectrometer. MaxQuant was used for label-free quantification [38], yielding a list of proteins normalized to total protein content (PROT). PROT/DW was quantified by dividing COL/DW from the hydroxyproline assay by COL/PROT from bottom-up proteomics (sum of all collagen proteins per PROT). Because day 1 and day 4 constructs disintegrated during the washing process, they were not included in the bottom-up proteomics analysis.

### Statistical analysis

Data from this study were analyzed using a one-way analysis of variance (ANOVA), with the only factor being culture time, followed by a post hoc Tukey’s honestly significant difference test performed in JMP Pro 14. All bar graphs were created in GraphPad Prism 9. A connecting letters report is used to show statistical significance in all bar graphs; bars that do not share the same letter are significantly different from each other.

## Results

### Neocartilage histology

**Fig. 2:**
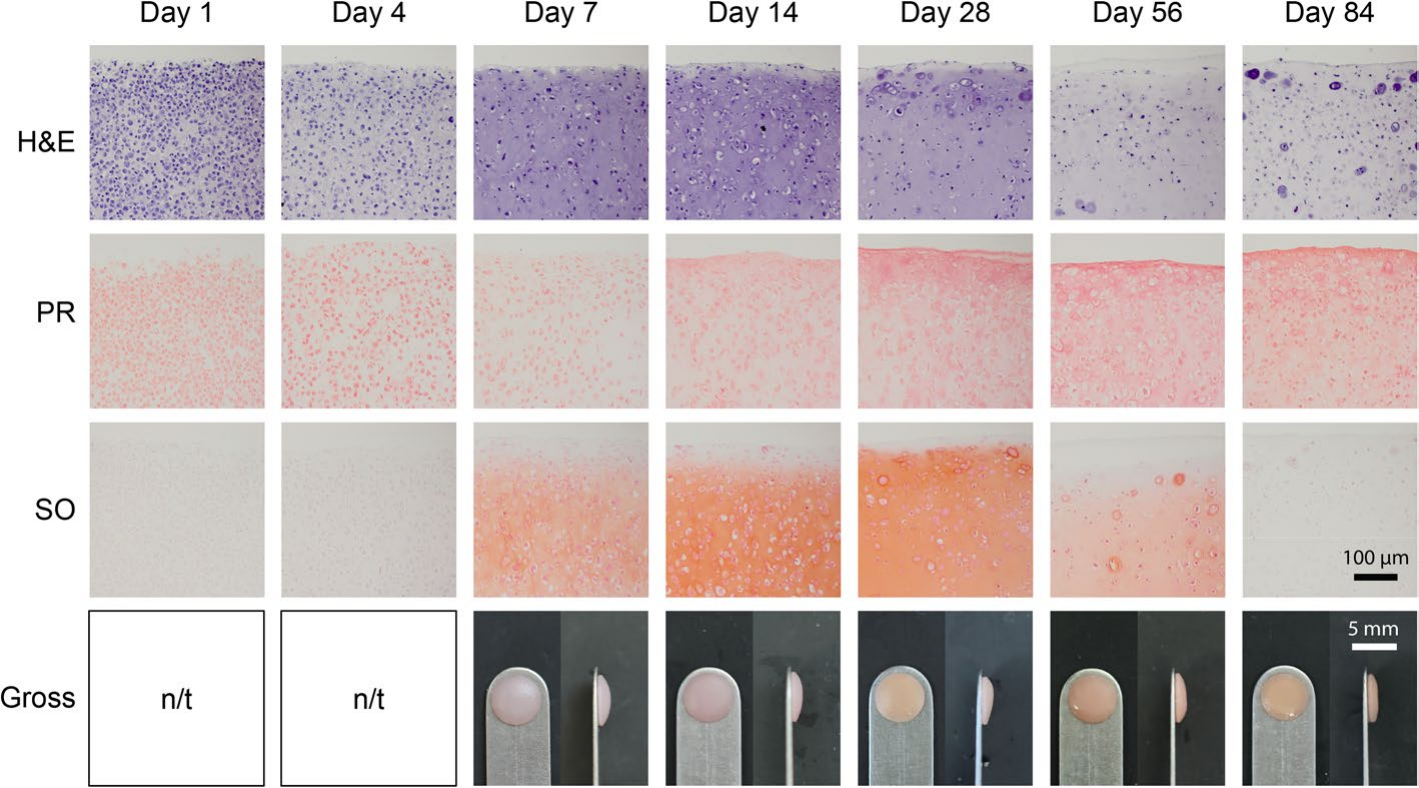
Histology and gross morphology of neocartilage constructs. Staining with hematoxylin and eosin (H&E), picrosirius red (PR), and safranin O with fast green counterstain (SO) is shown in self-assembled neocartilage constructs at different time points in culture. Gross morphology is shown in front and side views. Gross morphology pictures for day 1 and day 4 of culture were not taken (n/t)

Representative images for histology of H&E staining for cellular morphology, PR staining for collagen, and SO staining for GAGs, as well as gross morphology, are reported in Fig. 2. At earlier time points such as 1 and 4 days of culture, staining intensity is localized to the cells for both H&E and PR stainings. As the tissue matures to 7–28 days of culture, the staining becomes more intense for hematoxylin in the ECM of the neocartilage, but rapidly decreases after 56–84 days of culture, exhibiting almost no staining. However, the PR staining becomes more intense over time. SO staining follows a similar pattern to the H&E staining for 7–84 days of culture but yields minimal staining for the earlier 1- and 4-day time points. From 7 to 84 days of culture, constructs appear flat and robust.

### Mechanical properties

**Fig. 3:**
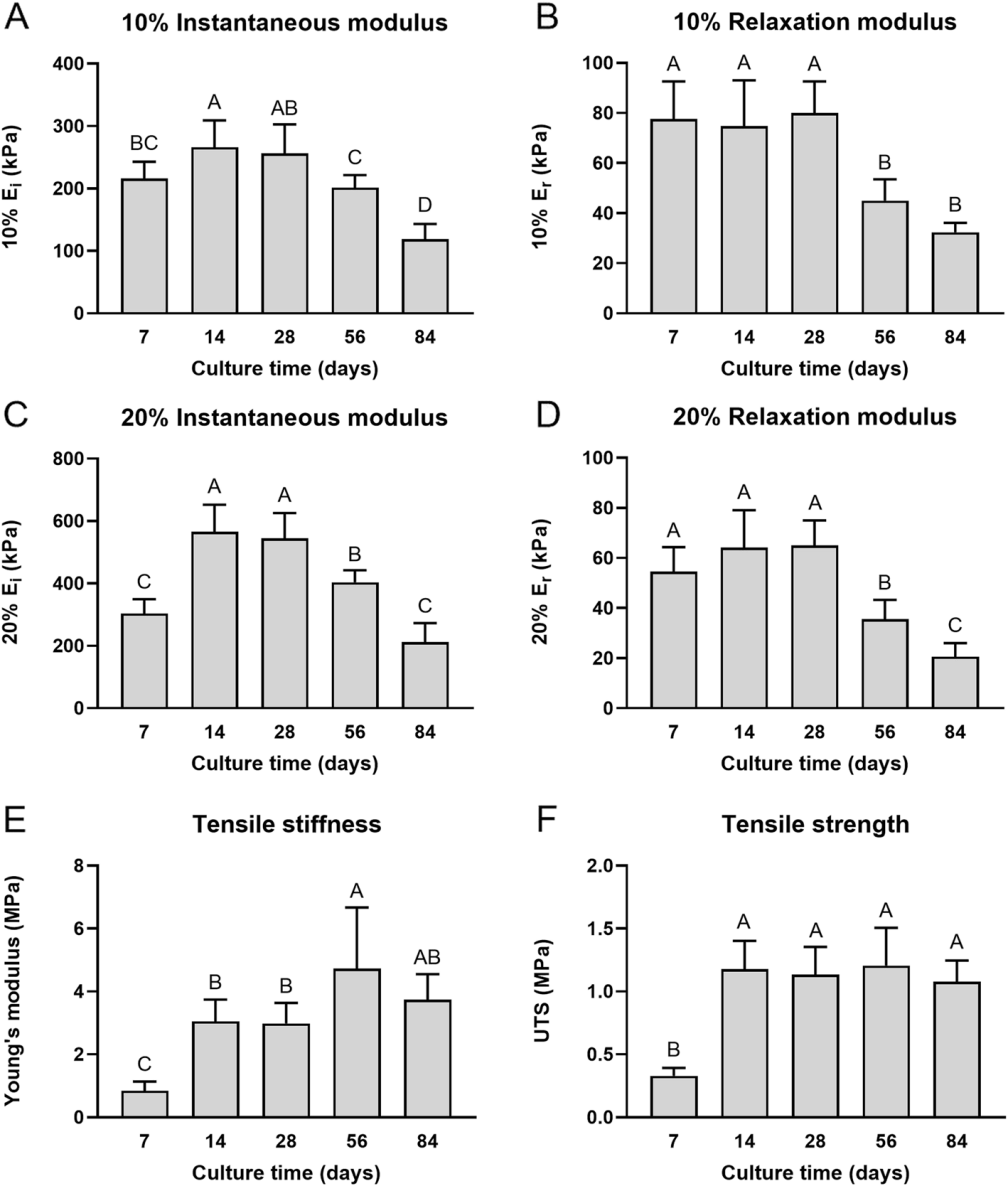
Mechanical properties of neocartilage constructs. At different time points throughout self-assembled neocartilage culture, compressive properties are shown in the 10% and 20% instantaneous modulus (E_i_) (**A**, **C**) and relaxation modulus (E_r_) (**B**, **D**) graphs. Tensile properties are shown in the Young’s modulus (**E**) and ultimate tensile strength (UTS) graphs (**F**). Bars that do not share the same letter are significantly different from each other

Mechanical properties from compressive stress-relaxation testing and uniaxial tension testing are reported in Fig. 3. Across culture times, instantaneous modulus for both 10% and 20% strain levels peaked at 14 days of culture. The maximum values for 10% and 20% instantaneous modulus obtained at 14 days were 266 ± 43 kPa and 565 ± 87 kPa, respectively, significantly higher than the values at both 7 days (216 ± 27 kPa for 10%, 304 ± 46 kPa for 20%, *p* < 0.05) and 56 days of culture (202 ± 20 kPa for 10% and 404 ± 39 kPa for 20%, *p* < 0.01); however, they were not different from 28 days of culture (Fig. 3A, C). For both 10% and 20% relaxation modulus, maximal points were observed at 28 days of culture, with significant decreases at 56 and 84 days (*p* < 0.0001) (Fig. 3B, D). Young’s modulus (4.7 ± 1.9 MPa) and UTS (1.2 ± 0.3 MPa) peaked at 56 days, 5.9-times and 4.0-times higher than their respective values of 0.8 ± 0.3 MPa and 0.3 ± 0.1 MPa at 7 days (Fig. 3E-F). Young’s modulus slightly decreased to 3.7 ± 0.8 MPa at 84 days of culture, which was 4.6-times higher than day 7, but the difference between 56 and 84 days of culture was not significant (Fig. 3E). UTS exhibited a significant increase from 7 to 14 days of culture (*p* < 0.0001), at which point it plateaued without any significant changes at any later time points (Fig. 3F). Additional outcomes including 10% and 20% coefficients of viscosity, strain at failure, and toughness are reported in Supplementary Table 1.

### Biochemical properties

**Fig. 4:**
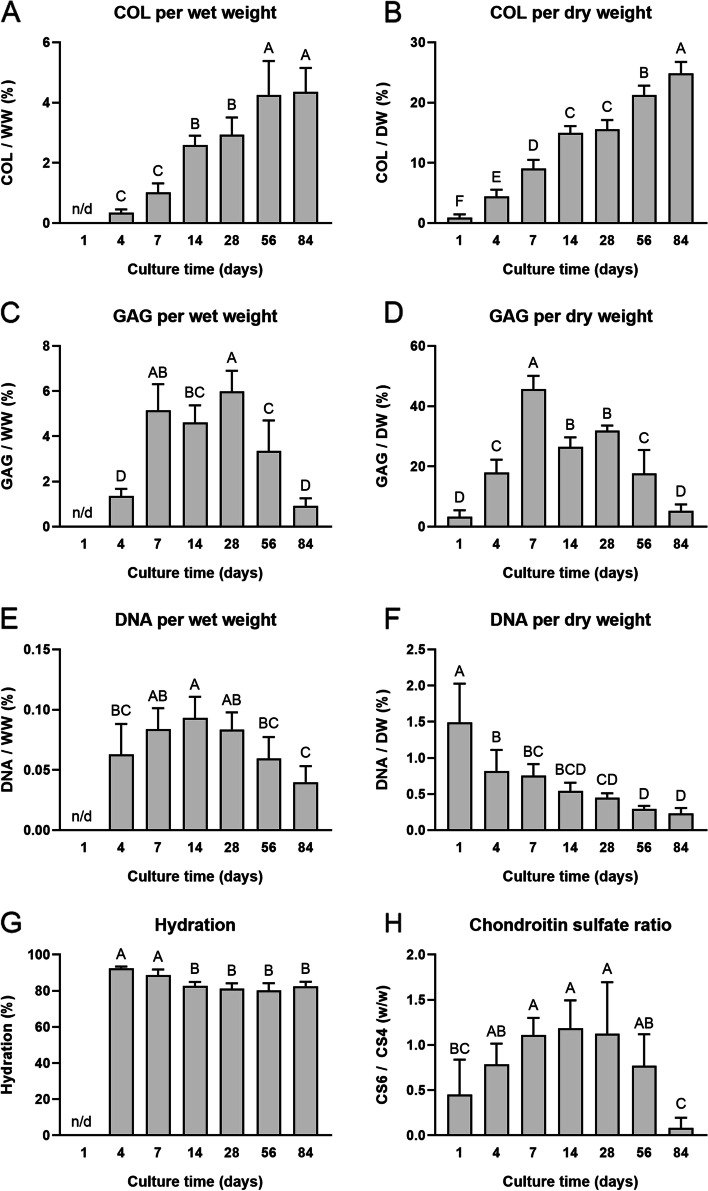
Biochemical composition of neocartilage constructs. At different time points throughout self-assembled neocartilage culture, the biochemical composition is shown. Total collagen (COL) content (**A**-**B**), glycosaminoglycan (GAG) content (**C**-**D**), and DNA content (**E**–**F**) are normalized to both wet weight (WW) and dry weight (DW). Hydration (**G**) is reported, along with the chondroitin-6-sulfate (CS6) to chondroitin-4-sulfate (CS4) ratio (**H**). Bars that do not share the same letter are significantly different from each other

For biochemical analysis of ECM content, COL, GAG, and DNA content are reported in Fig. 4. For COL/DW and COL/WW values, steady increases were observed over culture time, with the highest value seen at day 84 for both measurements (24.8 ± 1.9% and 4.4 ± 0.8%, respectively) (Fig. 4A-B). The COL/DW increased 27.6-times from day 1 to day 84 and 2.7-times from day 7 to day 84 (Fig. 4B). Interestingly, GAG/DW peaked at 7 days of culture (45.7 ± 4.4%), which was significantly higher than any other group (*p* < 0.0001) (Fig. 4D), while GAG/WW peaked at 28 days of culture (6.0 ± 0.9%) but was not significantly different from 7 days of culture (5.1 ± 1.2%) (Fig. 4C). By 84 days of culture, GAG content decreased toward those levels seen at 1–4 days of culture in both measures (Fig. 4C-D). DNA/DW also trended down with time, exhibiting an 84.6% decrease from days 1 to 84 and a 69.3% decrease from days 7 to 84 (Fig. 4F). Similarly, DNA/WW also significantly decreased between 14 days of culture to 84 days (*p* < 0.0001) (Fig. 4E). Generally, hydration also decreased with time, exhibiting a 9.6% decrease from 7 days of culture to 56 days (*p* < 0.0001) (Fig. 4G). The CS6:CS4 ratio rose until 14 days of culture (1.2 w/w) before exhibiting a stark and significant decrease between 56 and 84 days (0.8 ± 0.4 w/w to 0.1 ± 0.1 w/w, *p* < 0.01) (Fig. 4H).

### Crosslink quantification

**Fig. 5:**
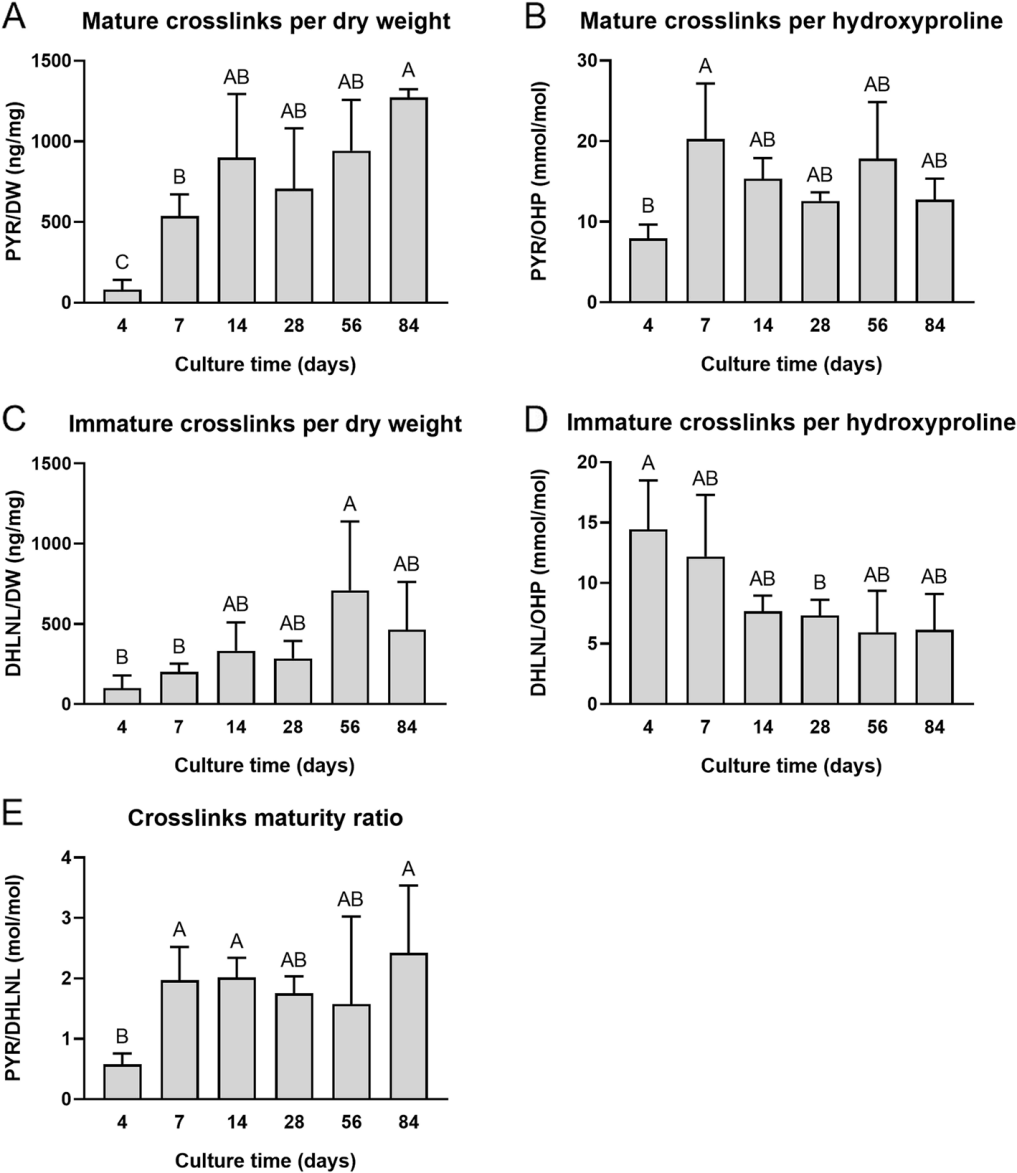
Collagen crosslink composition of neocartilage constructs. Throughout self-assembled neocartilage culture, mature pyridinoline crosslinks (PYR) and immature dihydroxylysinonorleucine crosslinks (DHLNL) are reported. PYR is normalized to dry weight (DW) (**A**) and hydroxyproline (OHP) (**B**). DHLNL is normalized to DW (**C**) and OHP (**D**). The PYR to DHLNL ratio is reported (**E**). Bars that do not share the same letter are significantly different from each other

Collagen crosslink analysis is reported in Fig. 5. PYR/DW was at a maximum after 84 days of culture, measuring 1273 ± 51 ng/mg (Fig. 5A). PYR/OHP significantly increased between 4 days (7.9 ± 1.7 mmol/mol) and 7 days (20.3 ± 6.9 mmol/mol) of culture (*p* < 0.01) but had no significant differences between days 7 and 84 (Fig. 5B). For DHLNL/DW, the values significantly increased over time from 100 ± 79 ng/mg after 4 days of culture to a maximum of 708 ± 430 ng/mg at 56 days of culture (*p* < 0.01) (Fig. 5C). The opposite trend was observed for DHLNL/OHP; between 4 days (14.4 ± 4.1 mmol/mol) and 28 days (7.3 ± 1.3 mmol/mol) of culture, there was a significant decrease in immature crosslinks (*p* < 0.05) (Fig. 5D). Overall, the maturity of the crosslinking within the constructs’ ECM increased, as depicted by the PYR/DHLNL ratio (Fig. 5E), significantly increasing 4.0-times from 4 days (0.6 ± 0.2 mol/mol) to 84 days of culture (2.4 ± 1.1 mol/mol, *p* < 0.05).

### Bottom-up proteomic analysis

**Fig. 6:**
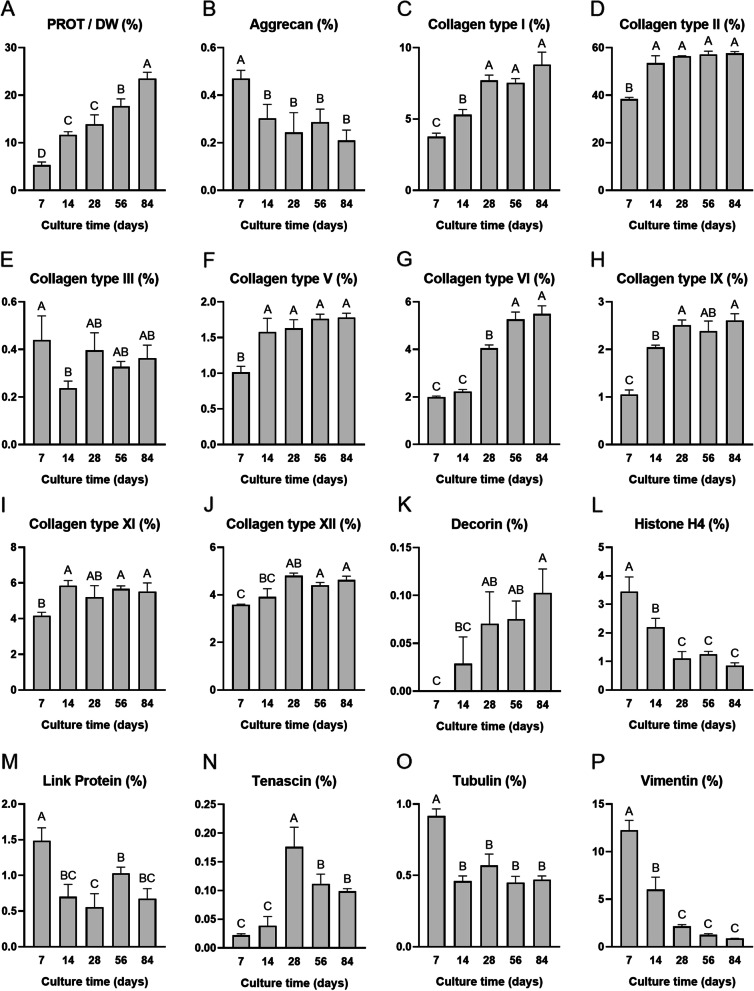
Bottom-up proteomics analysis of neocartilage constructs. Total protein (PROT) content (**A**) and 15 selected protein analytes of interest are reported. Graphs (**B**-**P**) are reported as percent protein per PROT. Bars that do not share the same letter are significantly different from each other

Bottom-up proteomics analysis identified and quantified a total of 364 protein analytes. Those with an intensity greater than 0.1%/PROT in at least one sample and all collagen chains (86 analytes total) are reported as averages in Supplementary Table 2. For post hoc analysis, 15 proteins of interest were chosen, and these data are reported in Fig. 6. Overall, PROT/DW significantly increased with culture time, rising 4.4-times from 7 days (5.30 ± 0.66%) to 84 days (23.50 ± 1.35%) of culture (*p* < 0.0001) (Fig. 6A). Similar trends were seen in collagen types I, II, V, VI, IX, XI, and XII, and decorin (Fig. 6C-D, F-K). For example, per PROT, collagen type II increased 1.5-times from 7 days (38.40 ± 0.74%) to 84 days (57.58 ± 0.81%) of culture (Fig. 6D). Aggrecan per PROT decreased over time, exhibiting a significant drop of 55.3% between 7 days (0.47 ± 0.03%) and 84 days (0.21 ± 0.04%) of culture (*p* < 0.01) (Fig. 6B). Link protein followed a similar trend (Fig. 6M). Most cell-associated proteins such as histone H4, tubulin, and vimentin all also decreased over time (Fig. 6L, O-P). Tenascin exhibited a parabolic-shaped trend, peaking after 28 days of culture (Fig. 6N). Collagen type X remained at levels below or equal to 0.05%/PROT for all culture time points (Supplementary Table 2).

## Discussion

The objective of this study was to characterize self-assembled neocartilage made from expanded, rejuvenated, self-assembled costal chondrocytes to determine if its development mirrors aspects of native hyaline cartilage. The hypotheses of this study were confirmed; self-assembled neocartilage derived from passaged, rejuvenated costal chondrocytes exhibited certain temporal trends in mechanics, biochemistry, and proteomics that were reminiscent of native hyaline cartilage development [27]. For example, when comparing juvenile to fetal porcine knee cartilage, increases in tensile properties, COL, and collagen type II, and decreases in DNA, aggrecan, and link protein have been noted [27]. Throughout maturation of self-assembled neocartilage, these same trends were mirrored with tensile properties, COL, and collagen type II increasing and DNA, aggrecan, and link protein decreasing during culture. Ultimately, this study 1) elucidated similarities in the ECM maturation of self-assembled neocartilage and native hyaline cartilage, 2) identified specific ECM components with quantities parallel to those in native hyaline articular cartilage and costal cartilage, 3) explored the proteomics of self-assembled cartilage ECM, including structure–function relationships and protein targets for future tissue-engineering techniques, and 4) established optimal time points for future implantation of self-assembled cartilage. Combined, these findings allow tissue engineers to identify targets and measures for potential quality control and release criteria for mechanically robust cartilage therapeutics, required for future preclinical and clinical studies.

The maturation of self-assembled neocartilage derived from expanded and rejuvenated costal chondrocytes followed mechanical trends of native hyaline cartilage development. Our group has recently published a mechanical, biochemical, and proteomic study of native porcine knee articular cartilage [27], which is used as comparison data for the neocartilage in this work; prior to this characterization, a comprehensive dataset of the developing native cartilage proteome did not exist. In native porcine knee articular cartilage, there was a 10.5-times increase in tensile Young’s modulus properties from the fetal to juvenile stage, then a slight decrease to mature tissue [27]. The neocartilage in this experiment exhibited a similar trend in tensile Young’s modulus, increasing 5.9-times from 7 to 56 days of culture, before a slight decrease at 84 days of culture. In compressive properties, similar trends applied to both native knee cartilage and self-assembled neocartilage. Native cartilage increased in compressive properties from the fetal to juvenile stages [27], and self-assembled neocartilage instantaneous moduli increased from 7 to 28 days of culture. A subsequent drop in compressive properties was seen in both native articular cartilage and neocartilage; the 20% relaxation modulus of native knee articular cartilage dropped 1.8-times from juvenile to mature [27], and, in neocartilage, this same property dropped 3.2-times from 28 to 84 days of culture. While mechanical properties are the primary design criteria for tissue-engineered cartilages, the biochemical and proteomic properties are also of crucial importance.

Biochemical and proteomic analysis of the neocartilage in this study also revealed many similarities to native articular cartilage development. In both native articular cartilage [27] and self-assembled neocartilage, COL content increased throughout development. More specifically and important to the function of hyaline articular cartilage, collagen type II increased over time in both knee cartilage [27] and neocartilage. However, the collagen subtype profiles in native tissue and neocartilage had some developmental differences; in native articular cartilage, collagen types I, VI, and XII did not significantly change with tissue age, while collagen types IX and XI decreased [27]. Interestingly, collagen types I, VI, IX, XI, and XII all increased throughout culture in neocartilage, highlighting the novel differences found through this bottom-up proteomic analysis. The collagen crosslink maturity ratio did not change throughout tissue development in native knee cartilage [27]. Here, reported for the first time in neocartilage constructs, the maturity ratio increased in neocartilage; this was likely due to medium supplementation of lysyl oxidase-like 2, an enzyme that catalyzes the production of mature collagen crosslinks [18, 39]. In terms of GAG content of native tissue, it decreased from neonatal to juvenile articular cartilage [27]. While this trend was seen in later time points of self-assembly (i.e., days 28 and beyond), earlier timepoints of self-assembly (i.e., days 1–14) exhibited rapid accumulation of GAG in the ECM. Similar to native knee cartilage [27], aggrecan and link protein, parts of hyaline cartilage’s proteoglycan structure, decreased in neocartilage throughout development. In agreement with a previous study on matrix maturation in self-assembled cartilage, the CS6:CS4 ratio decreases in later time points of self-assembly [10]. However, the decrease in the CS6:CS4 ratio is opposite to the trend previously shown in aging knee cartilage [27]. DNA and cellularity (from H&E staining) also decreased over time in neocartilage constructs, similar to the trends found in native tissue [27]. As expected, histone H4, tubulin, and vimentin decreased in parallel with cellularity due to their respective roles in chromatin and cytoskeletal structure, similar to native cartilage trends. Tenascin, previously associated with fetal articular cartilage development, decreases during later tissue maturation [40]. Thus, the increases seen here in tenascin in early stages of self-assembly may be correlated with the deposition of more ECM by the chondrocytes, but the subsequent decrease may be due to maturation of the neocartilage. Altogether, there were many mechanical, biochemical, and proteomic similarities between native articular cartilage development and culture of self-assembled neocartilage made from expanded, rejuvenated costal chondrocytes that were discovered in this study.

In this study, costal chondrocytes from the rib were intended for tissue-engineering of knee articular cartilage using an allogeneic, non-homologous approach (Fig. 1A). However, there were some specific analytes where the neocartilage is more reminiscent of donor costal cartilage than recipient knee cartilage. For example, collagen type I in neocartilage comprised 7.55–7.70%/PROT between days 28 and 56 of culture, which was similar to the collagen type I quantity in native porcine floating costal cartilage (6.69%/PROT) and is higher than in the femoral condyle (1.22%/PROT) [37]. Similarly, collagen type V in neocartilage (1.63–1.76%/PROT between 28 and 56 days of culture) was found to be in between the quantities reported for floating costal cartilage (3.13%/PROT) and femoral condyle articular cartilage (0.29%/PROT) [37]. Importantly and in contrast to other collagen subtypes which showed quantities similar to donor tissue phenotype, collagen type X, associated with hypertrophic and calcified cartilage [41, 42], remained at or below 0.05%/PROT in all neocartilage time points. The quantity of collagen type X in native floating costal cartilage was 0.98%/PROT, and, in native femoral condyle cartilage, it was less than 0.01%/PROT [37]. If there were an abundant presence of collagen type X, self-assembled neocartilage implants could potentially calcify, rendering them unsuitable for use in the knee. However, the self-assembling process was shown to change the costal chondrocyte phenotype toward that of articular chondrocytes and away from calcification as found in native costal cartilage. While future tissue engineering studies will need to address differences of donor and recipient tissues, the self-assembling process using costal chondrocytes produced a neocartilage that is suitable for non-homologous use in the knee.

Although this study quantifies specific protein analytes and compares them to both donor and recipient tissues for the first time, there are specific molecular pathways unique to the development of each native tissue that would also affect their use in tissue engineering approaches. For example, previous gene expression data from self-assembled neocartilage have shown increases in SOX9, ACAN, and COL2A1 expression after aggregate rejuvenation, which correspond with the development of native articular cartilage [30]. These mechanisms can be further investigated through a head-to-head tissue characterization using approaches such as RNA sequencing to understand the mechanisms behind the proteomic differences quantified here. When paired with proteomic data (e.g., the collagen type II and aggrecan quantification in this study), these mechanistic studies can describe the full picture of gene transcription and translation in developing tissues.

Bottom-up proteomics was used to quantify all proteins in developing neocartilages, giving insight to structure–function relationships and protein targets for future tissue-engineering studies. Well-documented structure–function relationships in articular cartilage predict a direct relationship between tensile properties and COL content [43], but the UTS in neocartilages plateaued after 14 days of culture while COL continues to increase throughout the entire culture time. Bottom-up proteomics may contribute new knowledge to the existing structure–function relationships and hint as to why this contradiction arises. For example, the overall collagen subtype profile became less abundant in collagen type II relative to the other collagens after day 14, where collagen type II plateaued and collagen types I, VI, IX, and XII continued to increase. Given the role of collagen types IX and XII in fibrillogenesis [44], it is possible that these other collagen types inhibited maturation of the collagen type II fibrils, and, thus, why tensile properties did not continue to increase after day 14. Collagen type IX was abundant in fetal knee cartilage (7.43%/PROT) but dropped in mature cartilage (0.80%/PROT) [27]. Interestingly, this drop seen in native tissue was not observed in neocartilage where collagen type IX started at 1.05%/PROT at day 7 of culture and increased to 2.61%/PROT at day 84. Unlike in native knee cartilage development, collagen type IX in neocartilage was not replaced by more collagen type II. Additionally, even though there was a small amount of collagen type XIV present in native articular cartilages (0.95%/PROT in fetal cartilage, < 0.01%/PROT in mature cartilage) [27], there was no detectable collagen type XIV in neocartilages. Contrastingly, collagen type XII was abundant in neocartilage constructs (3.59–4.80%/PROT) compared to native cartilages (0.15–0.35%/PROT), approximately a 10-times difference. Because both collagen type XII and XIV play similar roles in fibrillogenesis [44], the neocartilage may have been producing excessive collagen type XII as compensation for the lack of collagen type XIV. It would be of great interest to cartilage tissue engineers to determine novel mechanical or biochemical stimuli leading to the deposition of collagen type XIV. The field of tissue engineering will continue to benefit from bottom-up proteomic studies through deeper understanding of structure–function relationships and development of novel tissue-engineering strategies to target specific protein analytes, improving the functionality of engineered neotissues.

It is crucial to create neocartilages that can withstand the joint loading environment; thus, it is important to select an appropriate time of culture which maximizes neocartilage mechanical properties. For knee articular cartilage, the main form of loading is compression [43], and, thus, it is desired to implant neocartilage when it has maximal compressive properties. Here, we showed that both instantaneous and relaxation moduli reached their maximum around 28 days of culture and decreased at later time points, making 28 days the optimal time point for knee articular cartilage implantation. While articular cartilage functions under tensile stresses as well, the tensile magnitudes are not as large as those seen in fibrocartilages [14]. Tensile stiffness and strength increased beyond 28 days of culture, with 56 and 84 days of culture exhibiting the greatest tensile stiffness and strength. Thus, these later time points may also be considered for fibrocartilage therapeutics. In addition to tensile properties, collagen type I also increased significantly at 28–84 days of culture compared to earlier time points, further mimicking the biochemical makeup of fibrocartilages like the knee meniscus and temporomandibular joint disc. This study identified optimal culture times for neocartilage (i.e., 28 days for knee articular cartilage and 56–84 days for fibrocartilages) which will be important as this technology is translated toward preclinical and clinical studies.

## Conclusions

Tissue-engineered cartilage products are proceeding through the regulatory pipeline, with matrix-assisted autologous chondrocyte implantation already approved for use in the U.S. and many more in development [3]. Recent tissue engineering approaches, such as the developmentally inspired self-assembling process, have resulted in robust neocartilages that have functional properties similar to native cartilage. Through this study, we observed that neocartilage made from passaged, rejuvenated costal chondrocytes had many similarities in ECM development to native knee cartilage, as shown through mechanical, biochemical, and proteomic analyses. Optimal time points were identified to maximize compressive and tensile properties for eventual implantation into suitable large animal models for hyaline cartilage and fibrocartilage ailments. Through bottom-up proteomics it was shown that there were some similarities to donor costal cartilage, such as the presence of collagen type I, and some differences in ECM composition of native knee cartilage and tissue-engineered cartilage, such as the temporal trends of collagen types IX, XI, and XII. Importantly, collagen type X in the neocartilage was approximately 20-times lower than in native floating rib cartilage, supporting the non-homologous approach of using costal chondrocytes to produce neocartilages for the knee. This study is novel in that it is the first, to our knowledge, to analyze development of an engineered tissue with quantitative proteomics and to compare specific analytes to a target tissue, knee articular cartilage. Toward translation of engineered cartilages and other tissues, bottom-up proteomics should be considered for the study of structure–function relationships, development of quality control protocols, and creation of a multitude of release criteria. Because the ultimate goal of tissue engineering is to reach native tissue mimicry, bottom-up proteomics is a demonstrably powerful tool for investigating differences in, for example, native and engineered tissues. A deeper understanding of ECM composition will enable new tissue engineering strategies to recapitulate the unique biochemical and mechanical properties of native tissue, improving clinical outcomes for patients as tissue-engineered products undergo preclinical studies, clinical trials, and eventual widespread usage in humans.

## Supplementary Information

**Additional file 1. Supplementary Table 1:** Additional mechanical outcomes ofneocartilage constructs. Additional outcomes for compressive stress-relaxationand uniaxial tension tests are reported as mean ± standard deviation. Cells that do not share the same letter are significantly different from each other.

**Additional file 2. Supplementary Table 2:** Bottom-up proteomics analytes ofneocartilage constructs. All analytes are reported as a percentage per totalprotein (PROT) content. Of 364 identified analytes, those with an intensitygreater than 0.1%/PROT in at least one sample and all collagen chains (86 proteins total) are reported.

## Data Availability

The data that support the findings of this study are available from the corresponding author upon reasonable request.

## Abbreviations

OA: Osteoarthritis
ECM: Extracellular matrix
GAG: Glycosaminoglycan
DMEM: Dulbecco’s modified Eagle’s medium
FBS: Fetal bovine serum
PSF: Penicillin–streptomycin-fungizone
CHG: Chondrogenic medium
DMSO: Dimethyl sulfoxide
GDF-5: Growth differentiation factor 5
BMP-2: Bone morphogenetic protein 2
c-ABC: Chondroitinase ABC
WW: Wet weight
DW: Dry weight
H&E: Hematoxylin and eosin
UTS: Ultimate tensile strength
COL: Total collagen content
FACE: Fluorophore assisted carbohydrate electrophoresis
PYR: Pyridinoline
DHLNL: Dihydroxylysinonorleucine
OHP: Hydroxyproline
PROT: Total protein content
ANOVA: Analysis of variance
